# Variation suggestive of horizontal gene transfer at a lipopolysaccharide (*lps*) biosynthetic locus in *Xanthomonas oryzae *pv. *oryzae*, the bacterial leaf blight pathogen of rice

**DOI:** 10.1186/1471-2180-4-40

**Published:** 2004-10-09

**Authors:** Prabhu B Patil, Ramesh V Sonti

**Affiliations:** 1 Centre for Cellular and Molecular Biology (CCMB), Uppal Road, Hyderabad-500007, India

## Abstract

**Background:**

In animal pathogenic bacteria, horizontal gene transfer events (HGT) have been frequently observed in genomic regions that encode functions involved in biosynthesis of the outer membrane located lipopolysaccharide (LPS). As a result, different strains of the same pathogen can have substantially different *lps *biosynthetic gene clusters. Since LPS is highly antigenic, the variation at *lps *loci is attributed to be of advantage in evading the host immune system. Although LPS has been suggested as a potentiator of plant defense responses, interstrain variation at *lps *biosynthetic gene clusters has not been reported for any plant pathogenic bacterium.

**Results:**

We report here the complete sequence of a 12.2 kb virulence locus of *Xanthomonas oryzae *pv. *oryzae *(Xoo) encoding six genes whose products are homologous to functions involved in LPS biosynthesis and transport. All six open reading frames (ORFs) have atypical G+C content and altered codon usage, which are the hallmarks of genomic islands that are acquired by horizontal gene transfer. The *lps *locus is flanked by highly conserved genes, *metB *and *etfA*, respectively encoding cystathionine gamma lyase and electron transport flavoprotein. Interestingly, two different sets of *lps *genes are present at this locus in the plant pathogens, *Xanthomonas campestris *pv. *campestris *(Xcc) and *Xanthomonas axonopodis *pv. *citri *(Xac). The genomic island is present in a number of Xoo strains from India and other Asian countries but is not present in two strains, one from India (BXO8) and another from Nepal (Nepal624) as well as the closely related rice pathogen, *Xanthomonas oryzae *pv. *oryzicola *(Xoor). TAIL-PCR analysis indicates that sequences related to Xac are present at the *lps *locus in both BXO8 and Nepal624. The Xoor strain has a hybrid *lps *gene cluster, with sequences at the *metB *and *etfA *ends, being most closely related to sequences from Xac and the tomato pathogen, *Pseudomonas syringae *pv. *tomato *respectively.

**Conclusion:**

This is the first report of hypervariation at an *lps *locus between different strains of a plant pathogenic bacterium. Our results indicate that multiple HGT events have occurred at this locus in the xanthomonad group of plant pathogens.

## Background

LPS is an important constituent of the outer membrane of gram-negative bacteria. Variation in LPS composition can have profound consequences for these cells by potentially providing resistance against bacteriophages and antimicrobial compounds as well as facilitating evasion of the host immune system in animal pathogens. Extreme variation at LPS gene clusters has been reported in animal pathogenic bacteria. Recently, eleven highly divergent gene clusters were reported to occupy an LPSspecific locus in *Pseudomonas aeruginosa*, an opportunistic human pathogen [1]. The acquisition by horizontal gene transfer of a new LPS biosynthetic gene cluster in *Vibrio cholerae *is considered as a major cause for the cholera epidemic that originated in India in 1992 [2]. In plant pathogenic bacteria, LPS is an important virulence factor and mutations in the genes involved in LPS production result in severe virulence deficiency [3-8]. LPS has been shown to induce resistance in plants against pathogens [9,10] and in some recent studies, LPS is found to induce expression of plant defense genes [11,12] as well as an oxidative burst reaction in cell cultures [13]. Since LPS recognition appears to be an important aspect of plant defense responses, variation in *lps *gene repertoire is to be expected within different strains of plant pathogenic bacteria.

The genus Xathomonas includes a number of plant pathogenic bacteria. Two related members of this genus, *Xanthomonas oryzae *pv. *oryzae *(Xoo) and *Xanthomonas oryzae *pv. *oryzicola *(Xoor) cause diseases of rice [14]. They exhibit different tissue specificities with Xoo growing in the xylem vessels while Xoor grows within the intercellular spaces of the parenchymatous tissue. Xoo causes bacterial leaf blight, the most serious bacterial disease of rice. This disease is prevalent in many rice growing countries in Asia, extending from the Indian subcontinent to Japan and Korea. DNA fingerprinting studies using multi-locus RFLP and PCR probes have indicated that there is extensive genetic diversity within Xoo strains isolated from various countries [15-19]. In India, multi-locus RFLP profiling has indicated that one lineage of Xoo (called the BXO1 lineage, based on the type strain for this group) is widely distributed within the country. Strains within the BXO1 lineage cluster together at about the 90 % similarity level in a dendrogram. A second group of strains is quite diverse, both at the haplotypic and pathotypic level, and clusters with the BXO1 group at about the 55% similarity level [19].

In previous research, we have reported a 5.5 kb region in the genome of Xoo strain BXO1 and demonstrated that it encodes three genes that are involved in biosynthesis of LPS and extracellular polysaccharide (EPS) as well as in virulence [8]. All the three genes have atypical G+C content, as compared to the rest of the Xoo genome. In this study, we have completed the entire sequence of this 12.2 kb genomic locus and indicate that it encodes three additional genes, *wxoD*, *wzt *and *wzm*, that are postulated to be involved in LPS biosynthesis and transport. These newly described genes also have atypical G+C content and all the six genes at this locus exhibit altered codon usage pattern, as compared to other Xoo genes. We present evidence that this locus is present in many, but not all, Xoo strains and that it is absent in Xoor. Our results indicate that there is substantial variation at this locus among various xanthomonads. The possible significance of these results is discussed.

## Results

### Genetic organization of a Xoo *lps *locus

**Figure 1:**
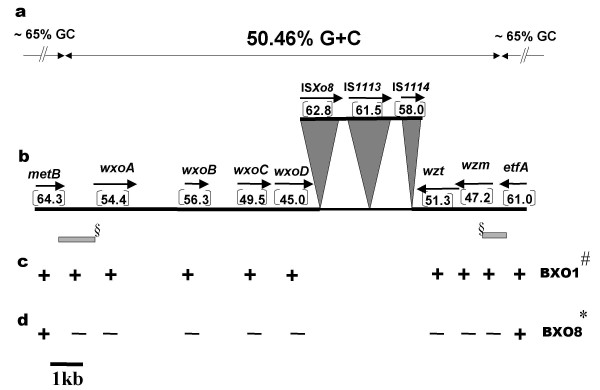
**Genetic organization of a locus encoding LPS biosynthetic genes in Xoo strain BXO1. **a. Overall G+C content of the locus and the flanking regions. The G+C content of the genomic island was calculated without including the sequences of IS elements. The overall G+C content of the genome is ~65%. b. Organization and G+C content of individual genes and transposases of IS elements. IS*1114 *encodes a truncated ORF. Arrows indicate transcriptional orientation. c. and d. Presence (+) and absence (-) of genes/PCR products in particular strains. ^§ ^Indicates PCR products obtained using primer pairs directed against either *metB *and *wxoA *or *etfA *and *wzm*. ^# ^similar results were obtained with all Xoo strains tested excepting BXO8 and Nepal 624. * similar results were obtained with the Nepal 624 strain.

In an earlier study, a novel Xoo locus was reported to be required for LPS and extracellular polysaccharide (EPS) production as well as virulence. A 35 kb cosmid, pSD5, that complements mutations in this region was isolated [8]. Partial sequence (5.5 kb) of this locus indicated that the region has atypical G+C content and contains three genes which encode a predicted sugar nucleotide epimerase and two predicted glycosyl transferases. We report here the complete 12.2 kb sequence and genomic organization of this locus in Xoo strain BXO1 (Fig. 1). The insert in the pSD5 cosmid includes 7 *Eco*RI fragments (0.6, 2.2, 3.5, 4.0, 6.0, 9.0 and 10 kb). We subcloned all the fragments into pBlueScript. Based on the end sequences of the inserts in the subclones and pSD5, the *lps *locus was mapped to four of these *Eco*RI fragments (0.6, 4, 3.5 and 9 kb). The previously obtained sequence was found to include all of the 3.5 kb and part of the 4 kb fragment and the remaining sequence of this region was obtained by sequencing the 0.6 kb and the 9 kb fragment (Please refer Methods). A total sequence of 13.18 kb was constituted by joining 6.14 kb of previously obtained sequence [8] and 7.04 kb of new sequence. The 13.18 kb sequence includes 12.2 kb of the *lps *locus and some flanking regions. The additional sequence of the *lps *locus encodes three putative genes which encode a predicted O-antigen acetylase, a predicted ABC transporter permease and a predicted ATP-binding protein and three insertion sequence (IS) elements.

All of the putative genes have been named as per Bacterial Polysaccharide Genes Nomenclature (BPGN) [20]. The first three genes, *wxoA *(encodes a predicted epimerase), *wxoB *and *wxoC *(both encode predicted glycosyl transferases) have been described earlier. The fourth gene is *wxoD *and encodes a predicted 327 amino acids long protein. A BLAST [21] search reveals strong homology to acetyltransferases that are involved in LPS modification and the best match is with an acetyltransferase from *Mesorhizobium loti *(MAFF303099; 34% identity and 46% similarity at amino acid level). Interestingly, no homologs of this gene have been reported in any other xanthomonad. The fifth gene, *wzt*, encodes a predicted 436 amino acid long protein. A BLAST search reveals homology to functions involved in LPS transport. The best match is with the ATPase component of an ABC-type polysaccharide transport system from *Burkholderia fungorum *(ZP_00033174.1; 47% identity and 65% similarity at amino acid level). The sixth gene, *wzm*, encodes a predicted 437 amino acid long protein which is homologous to integral membrane protein components of ABC transporter systems that are involved in LPS transport. The best match is with a permease component of the ABC-type polysaccharide export system from *Pseudomonas fluorescens *PfO-1 (ZP_00085342.1; 50% identity and 65% similarity at amino acid level). The start codon of *wzt *overlaps with the stop codon of *wzm*. Homologs of *wzt *and *wzm *are typically present in many *lps *gene clusters. Interestingly, two complete Insertion Sequence (IS) elements (IS*Xo8 *and IS*1113*) and one truncated IS element (IS*1114*) interrupt this cluster between the genes, *wxoD *and *wzt*. IS*Xo8 *is a novel 1320 bp long insertion sequence and a BLAST search shows homology to transposase of ISRSO*17 *encoded by *Ralstonia solanacearum *(CAD17626; 51% identity and 63% similarity at amino acid level). A complete copy of the IS*1113 *element (AF482989) and a truncated copy of the IS*1114 *element (AF232058) are also present as indicated in Fig. 1. The presence of IS elements is a marked feature of many *lps *loci [22]. Transcriptional orientation suggests the possibility that ORFs *wxoA*, *wxoB*, *wxoC *and *wxoD *might constitute one operon and that ORFs *wzm *and *wzt *might be transcribed together. The overlap between the start codon of *wzt *and the stop codon of *wzm *also suggests that these two genes are co-transcribed.

The *lps *locus is flanked by *metB*, which encodes a predicted cystathionine gamma lyase, and *etfA *which encodes a predicted electron transport flavoprotein. The genome sequences of *Xanthomonas campestris *pv. *campestris *(Xcc; infects crucifer plants like cabbage, cauliflower, mustard, etc.) and *Xanthomonas axonopodis *pv. *citri *(Xac; infects citrus plants) have been obtained [23]. The Xoo *metB *gene (a partial sequence of 642 bp is available) exhibits within the sequenced region, 91% and 88% nucleotide identity to *metB *genes of Xac (AE012010.1) and Xcc (AE012157.1), respectively. The Xoo *etfA *gene (a partial sequence of 328 bp is available) exhibits within the sequenced region, 93% and 91% nucleotide sequence identity, respectively, with *etfA *genes in Xac (AE012009.1) and Xcc (AE012159.1). Interestingly, the *lps *biosynthetic gene cluster of Xcc, which comprises fifteen genes, is also located between the *metB *and *etfA *genes [24]. In Xac, this gene cluster is missing at this locus and is replaced by a set of fourteen genes, several of which are homologous to functions involved in LPS synthesis and transport. The gene clusters present at this locus in Xcc, Xac and Xoo have distinct nucleotide sequences, gene numbers (15 genes in Xcc, 14 genes in Xac, 6 genes in Xoo) and gene organization.

### The Xoo *lps *cluster is a genomic island

#### a) Atypical G+C content

The average G+C content of Xoo and other Xanthomonads is estimated to be around 65% [25], while the average G+C content of the *lps *locus is 50.46% (excluding the IS elements) [Fig. 1]. The variation is much more marked among the genes, from as low as 45.0% (*wxoD*) to 56.3% (*wxoC*). Atypical G+C content is a characteristic feature of "genomic islands" that are believed to be acquired by horizontal gene transfer. The transposase genes encoded by IS*Xo8 *and IS*1113 *have a G+C content that is >61%, a value which is typical for the genomes of Xoo and other xanthomonads. The G+C content of *metB *and *etfA *genes that flank the genomic island have G+C content of 64.3% and 61% respectively (within the partial sequences that have been obtained) which is typical of the Xoo genome.

#### b) Altered codon usage

**Figure 2:**
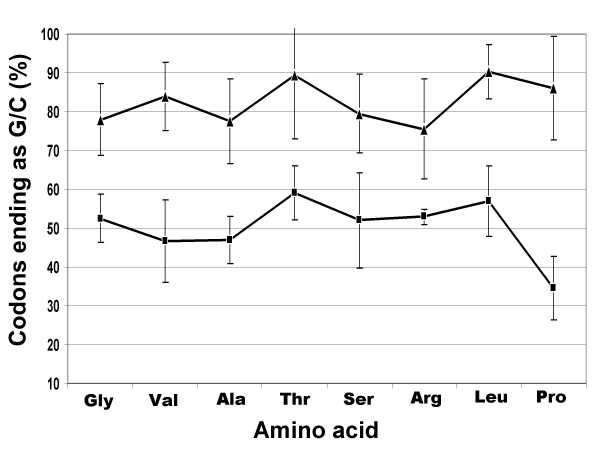
**Genes encoded in the *lps *locus exhibit altered Codon Usage Pattern (CUP). **Eight amino acids, each of which has atleast four synonymous codons, are represented on the X-axis. The % of codons ending with G/C for each of these amino acids is represented on the Y-axis as mean ± SD. The lower line represents CUP for eight aminoacids of the six genes (excluding transposase ORFs) encoded in the *lps *locus. The upper line represents CUP of six Xoo genes from elsewhere in the genome (Please refer Methods).

An additional hallmark of a genomic island is the altered codon usage. Here we present a simple and graphical way of calculating and representing the codon usage differences and refer to it as Codon Usage Pattern or CUP (Please refer Methods). Eight aminoacids, i.e., Glycine, Valine, Threonine, Leucine, Arginine, Serine, Proline and Alanine, were selected to study CUP because they have atleast four synonymous codons. The percentage of synonymous codons that end with G or C was calculated for each aminoacid and gene. This analysis was conducted for six genes of the *lps *island and six genes from elsewhere in the Xoo genome (please refer Methods). We show that CUP of the genes present in the genomic island is dramatically different from the typical Xoo genes (Fig. 2). The %G+C at third codon position of synonymous codons for amino acid Glycine is only 52.5 % for genes present in the *lps *locus, while it is 78 % in case of Xoo genes that are located elsewhere in the genome. Similarly, for amino acids Valine, Alanine, Threonine, Serine, Arginine, Leucine and Proline the values are 46.6, 47, 59, 52, 53, 57 and 34.6 % respectively for genes at the *lps *locus, while the values are 84, 77.5, 89.5, 79.5, 75.6, 90.3 and 86.16 % for the respective aminoacids in case of the typical Xoo genes. Altered codon usage is a characteristic feature of horizontally acquired genes and CUP clearly indicates that the Xoo *lps *cluster is a genomic island (Fig. 2).

### The *lps *locus is present in the genomes of many, but not all, Xoo strains

**Figure 3:**
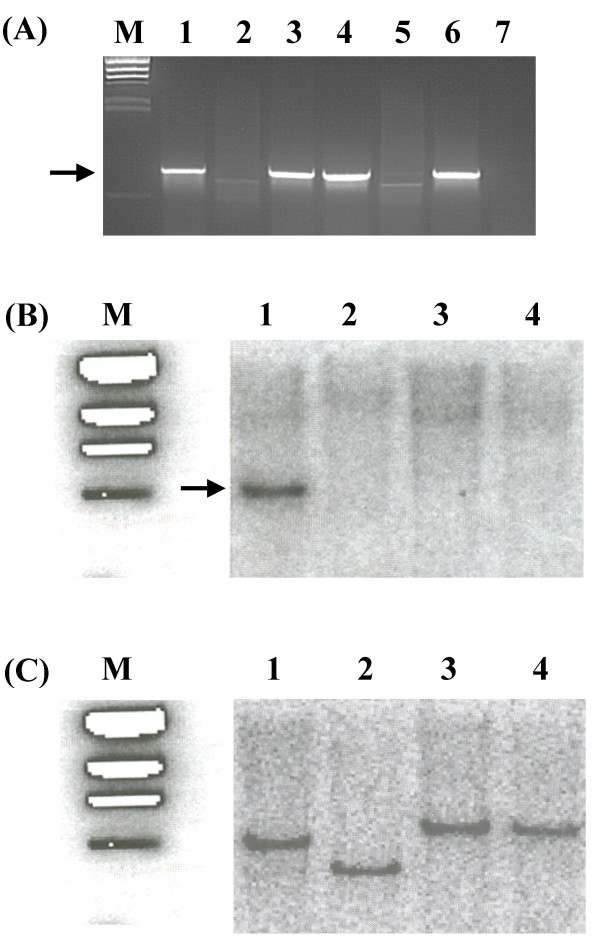
**The *lps *locus is absent from the genomes of Xoo strains BXO8, Nepal624 and Xoor strain BXORI. **(A) PCR analysis using primers that are specific to *wxoA *gene. M is the λ *Hind*III Marker lane. An expected band of 1 kb (indicated by arrow) is present in the Xoo strains, BXO1 (lane 1), BXO5 (lane 3), BXO6 (lane 4) and BXO20 (lane 6) but absent in BXO8 (lane 5), Nepal624 (lane 7) and BXORI (lane 2). (B) Southern hybridization analysis of *Eco*RI digested genomic DNA using α-^32^-P labeled *wxoA *specific probe (see Methods). A 4 kb band can be seen (indicated by arrow) in BXO1 (lane 1) but not in BXORI (lane 2), BXO8 (lane 3) and Nepal624 (lane 4). Similar results were obtained for *wxoB*, *wxoC*, *wxoD*, *wzm *and *wzt *genes. (C) The blot from (B) was deprobed and was hybridized with α-^32^P labeled probe specific to the *metB *gene. A specific band can be seen in all the lanes. Note the sizes of the bands indicating that *metB *is present in different *Eco*RI fragments in BXO1, BXORI and BXO8/Nepal624.

The presence of the genomic island in different Xoo strains was assessed by PCR using gene specific primers, for all the six *lps *genes, as described in the Methods. The list of strains used in the study is given in the Table 1 and the list of gene specific primers is given in Table 2. In order to confirm that the genomic island is present at the same genomic location in all strains, PCR was also performed using two primer pairs that are designed to amplify fragments from *metB *to *wxoA *and *wzm *to *etfA*, respectively. The analysis included nine Indian Xoo strains representing different geographic locations and the BXO1 and non BXO1 groups. The list also includes twelve Xoo strains from different Asian countries and a Xoor strain, BXOR1, from India. Our study revealed that the genomic island is present in the majority (7/8) of Xoo strains that we have examined from India (Fig. 1). Four BXO1 group strains (BXO4, BXO7, BXO13 and BXO479) and three of the non-BXO1 strains (BXO5, BXO6 and BXO20) have the genomic island. The genomic island is also present in two strains each from China, Malaysia, Indonesia, Philippines, Korea and one strain from Nepal (Fig. 1, Table 1). The *lps *locus is present, in all these strains, between the *metB *and *etfA *genes. Interestingly, we find that the genomic island is not present (as judged by PCR [Fig. 3A] and Southern hybridisation [Fig. 3B]; see Methods) in the genomes of Xoo strains BXO8 and Nepal624, as well as the Xoor strain, BXORI. The results obtained with the probes directed against the *wxoA *gene are presented but similar results were obtained using probes that are specific for the other five genes. The blots used above were reprobed as a positive control with a *metB *specific probe and the results gave an expected size band in BXO1 (lane 1), and different sized bands in BXO8 (lane 3), Nepal624 (lane 4) and BXOR1 (lane 2) indicating that the *metB *gene is present but located in different *Eco*RI fragments (Fig. 3C).

### BXO8 and Nepal 624 have sequences related to Xac at the *lps *locus

What are the sequences present at this genomic location in the Xoo strains that lack the *lps *locus? Thermal Asymmetric Interlaced (TAIL) PCR is an efficient technique for isolation of target DNA segments adjacent to known sequences [26]. TAIL-PCR and sequencing using primers directed against the conserved flanking *metB *and *etfA *genes suggests that sequences which are significantly similar to the Xac *lps *gene cluster are present at this genomic location in both of these strains. Next to *metB*, a *wzm *homolog is present in BXO8 (a partial sequence of 398 bp is available) and Xac with 69.2% identity at nucleotide level within the sequenced region. Next to *etfA*, a putative integral membrane protein encoding gene is present in both BXO8 (a partial sequence of 405 bp is available) and Xac with 91.3% identity at nucleotide level within the sequenced region. The BXO8 and Nepal624 strains exhibit 100% nucleotide sequence identity within the sequenced region. TAIL- PCR analysis of the Xoor strain indicates that it has a hybrid *lps *gene cluster. Next to *metB*, a unique *wzm *gene is located (a partial sequence of 548 bp is available) which exhibits 62.8% nucleotide identity to *wzm *gene of *Pseudomonas syringae *pv. *tomato *strain DC3000 (AE016859.1). Next to *etfA*, a putative inner membrane protein encoding gene is located (a partial sequence of 402 bp is available) which exhibits 97% and 92% nucleotide sequence identity, respectively, with similarly located genes in BXO8 and Xac. Because the BXO8 and Nepal624 strains have different sequences at the *lps *locus, as compared to other Xoo strains, we inoculated these strains along with appropriate controls onto leaves of the susceptible rice cultivar Taichung Native-1. We find that BXO8 and Nepal624 strains are able to cause typical bacterial leaf blight disease symptoms that are indistinguishable from those elicited by other Xoo strains (data are not shown).

### Presence of inverse repeats at the 3' ends of *metB *and *etfA *genes that flank the *lps *locus

**Figure 4:**
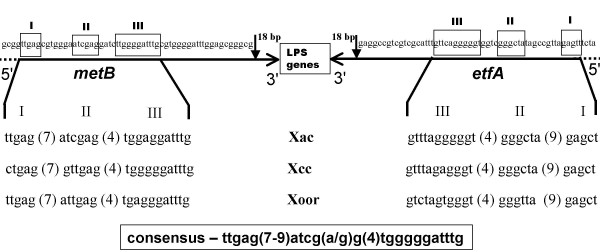
**Invert repeat sequences flanking the *lps *gene cluster in the BXO1 strain of Xoo. **The horizontal arrows represent the ORFs of *metB *and *etfA*. I, II, III represent three different invert repeats in the 3' regions of *metB *and *etfA *genes. The vertical arrows represent the breakpoints of homology between BXO1 and BXO8. The distances of the break points from the stop codons of the *metB *and *etfA *genes are indicated. Dashed lines indicate the remainder of the *metB *and *etfA *genes. The sequence of the corresponding inverse repeats in Xac, Xcc and Xoor are also indicated along with the derived consensus sequence for the repeats. The numbers in brackets indicate distances in bp between individual repeats.

We have performed an alignment using BLAST2 [27] of the nucleotide sequences derived from the *metB *and *etfA *genes in BXO1 and BXO8. The homology breakpoints appear to localise to the 3' regions of *metB *and *etfA *genes, exactly 18 bp upstream of their respective stop codons. Upto the break points, within the sequenced region at either end of the *lps *locus, the nucleotide sequence is identical in BXO1, BXO8 and Nepal624. The DNA sequence immediately preceding the break points was examined manually for presence of direct or inverse repeats. Interestingly, we could find three inverted repeats (I, II and III) within the 3' regions of *metB *and *etfA *near the homology breakpoints between BXO1 and BXO8 (Fig. 4). The first repeat is the smallest one (5 bp) and the third repeat is the largest (11 bp). The second repeat is 6 bp long and is 7 bp from the first repeat on the *metB *side and 9 bp from the first repeat on the *etfA *side. The distance between the second and third repeats is 4 bp in *metB *and *etfA*. We also found similarly located inverse repeats in the *metB *and *etfA *genes of Xac, Xcc and Xoor. A consensus sequence of the repeats was derived (Fig. 4) by scoring a nucleotide if it is present in a majority of repeats.

### Relationship between BXO8 and Nepal 624 strains

**Figure 5:**
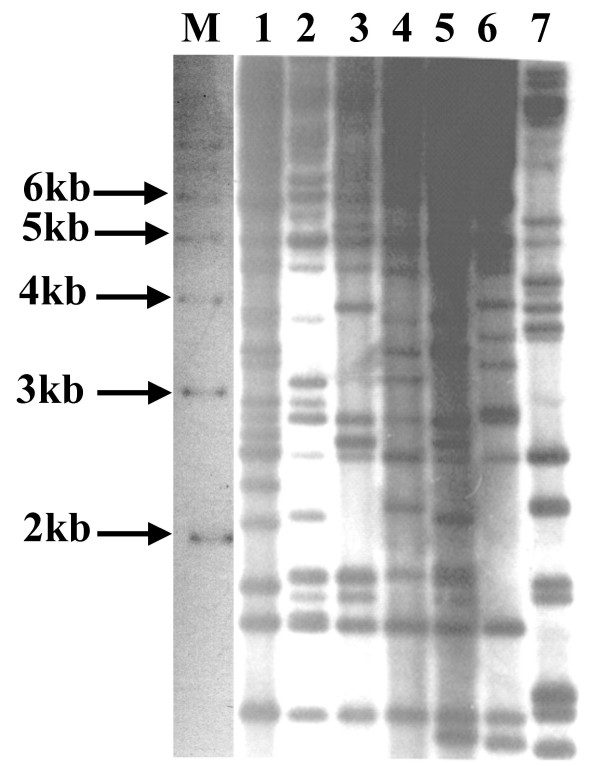
**Restriction fragment length polymorphism analysis of Xoo strains. **Southern analysis of *Eco*RI-digested genomic DNA was performed using α-^32^P labeled IS*1112 *as a probe (see Methods). Lanes: 1; BXO1, 2; BXO5, 3; BXO6, 4; BXO8, 5; BXO20, 6; Nepal624, 7; Xoor strain BXORI. M; indicates the size of molecular weight markers in kb.

**Figure 6:**
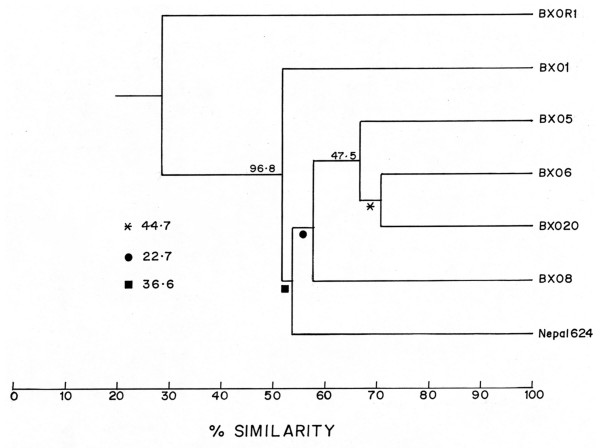
**Cluster analysis of Xoo strains. **The dendrogram was constructed using the UPGMA option of PHYLIP on the basis of restriction fragment length polymorphism data obtained with IS*1112 *probe. Numbers or symbols at the internal branches indicate bootstrap values for clusters. The BXORI (Xoor) strain constitutes the outgroup.

The TAIL PCR results indicate that the BXO8 and Nepal624 strains have identical sequences in place of the BXO1 *lps *locus. As both the strains are from the Indian subcontinent, there is the possibility that these are identical/nearly identical to each other. We therefore performed DNA fingerprinting analysis of the BXO8 and Nepal624 strains using the IS*1112 *insertion element as a probe. This probe is highly informative and can clearly differentiate the BXO1 and non BXO1 group of strains in India [19]. The following strains were also included in the analysis: BXO1, three non BXO1 group strains (BXO5, BXO6, BXO20) and BXORI. The hybridisation pattern revealed that BXO8 and Nepal624 are quite distinct from each other (Fig. 5). We could score 42 unique bands and the data generated were used to calculate pairwise similarity coefficients and cluster analysis was performed to generate a dendrogram using UPGMA (please refer Methods). The similarity coefficient between BXO8 and Nepal624 is only 56%. The dendrogram (Fig. 6) indicates that BXO8 clusters with BXO#s 5, 6 and 20 at about the 58% similarity level while Nepal624 clusters with all these four strains at about the 53% similarity level. All of the Xoo strains cluster with each other at about the 51% similarity level. Although the bootstrap values for these clusters are low, it is clear that the BXO8 and Nepal624 strains are not closely related to each other. As expected for an outgroup strain, BXOR1 clusters with Xoo strains at the 29% similarity level and the bootstrap value for this cluster is a high 96.8%.

## Discussion

We report here the complete sequence and genomic organization of the *lps *locus in the BXO1 strain of Xoo. Three of the genes in this locus i.e., *wxoA*, *wxoB *and *wxoC *were shown in an earlier study to be required for lipopolysaccharide production and virulence [8]. The predicted proteins encoded by the three new genes i.e., *wxoD*, *wzt *and *wzm *described in the present study are homologous to functions involved in lipopolysaccharide modification and transport. The *wxoD *gene encodes a predicted O-antigen acetylase which is homologous to similar functions encoded in phage genomes and other bacteria. O-antigen is the most variable part of LPS. Acetylation of O-antigen is shown to confer resistance to anitimicrobial peptides in *Proteus mirabilis *[28] and determines serotype in many bacterial pathogens [29-31]. The other two genes, *wzm *and *wzt*, are typically present in most *lps *gene clusters [including those of Xac and Xcc][23] as tandem genes and encode functions involved in LPS transport. The *wzm *and *wzt *genes of BXO1 have overlapping ORFs, an arrangement that is also seen in *wzm *and *wzt *genes of the *lps *loci in other bacteria including Xac. IS elements are frequently found interrupting many *lps *loci [22] and in BXO1, three IS elements interrupt the gene cluster between *wxoD *and *wzt *genes.

The complete genome sequences of more than 150 bacteria are now available [32] and studies have revealed the presence of DNA segments with G+C content and codon usage different from the rest of the genome. These regions are referred to as genomic islands and are believed to be acquired by horizontal gene transfer [33,34]. Another feature of genomic islands is their absence from the genomes of closely related strains. Our study clearly indicates that the *lps *locus of Xoo strain BXO1 fulfils all of the above criterion and constitutes a genomic island. The G+C content of this *lps *locus, excluding the IS elements, is 50%. The transposases encoded by IS*Xo8 *and IS*1113 *have a G+C content that is >61%. This value, which is typical for the genomes of Xoo and other xanthomonads [25], suggests the possibility that these elements have transposed into the *lps *locus after it's transfer into the Xoo genome. The presence of this genomic island in Xoo strains that are distributed across a vast segment of the Asian continent suggests that it was introduced into the Xoo genome early in the evolution of this pathogen.

**Figure 7:**
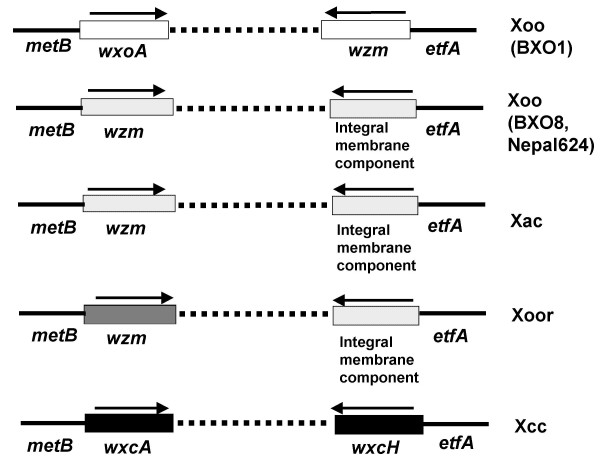
**Variation in *lps *gene clusters within the xanthomonads. **The genes that are adjacent to *metB *and *etfA *in different xanthomonads are indicated. Dashed lines represent the remainder of the *lps *cluster. Empty and filled boxes represent sequences specific to Xoo and Xcc respectively. Boxes with dots indicate that the sequences are either from or related to Xac genes. Box with stripes represents sequences that are related to *Pseudomonas syringae *pv. *tomato*. Arrows indicate transcriptional orientation. The w*zm *gene encodes a predicted ABC transporter permease protein, *wxoA *encodes a predicted epimerase, *wxcA *encodes a glycosyl transferase and *wxcH *encodes a hypothetical protein.

The BXO8 and Nepal624 strains do not have the *lps *locus that is present in the other Xoo strains. The related xanthomonad, Xoor, also has an *lps *locus that is different from the BXO1 *lps *locus. Also, different gene clusters are present at this locus in Xac and Xcc (Fig. 7). This indicates that multiple HGT events have occurred at this locus among xanthomonads. One HGT event occurred early in (or possibly at the time of) the evolution of the Xoo pathogen. This led to the introduction of the genomic island described in Figure 1. Two separate HGT events are likely to have occurred in the lineages that gave rise to BXO8 and Nepal624 Xoo strains. This is inferred from the observation that BXO8 and Nepal624 are quite unrelated in their genomic background. Another HGT can be inferred to have occurred in the Xoor strain wherein sequences that are most closely related to *Pseudomonas syringae *pv. *tomato *have been introduced at one end of the *lps *cluster. At least one more HGT has occurred to differentiate the *lps *gene clusters in Xcc and Xac.

The presence of invert repeats in the regions that flank the *lps *locus is likely to be significant. The presence of these repeats in the *metB *and *etfA *genes is especially striking as both genes encode completely different functions. The location of the repeats flanking the Xoo *lps *locus suggests that they might be involved in promoting recombination during HGT and/or gene regulation. A short inverted repeat sequence (GGCCAATCGA) flanking the lipopolysaccharide gene cluster has been reported in *Mycobacterium avium *subsp. *paratuberculosis *[35]. Another conserved sequence, called JUMPstart has been found located in intergenic regions upstream of polysaccharide biosynthetic gene clusters in several animal pathogenic bacteria like *Escherichia coli *strain K5, *Vibrio cholera*, etc. This sequence was implicated to be involved in gene regulation and has also been suggested to have a role in recombination [22,36].

As LPS is highly immunogenic, *lps *loci of animal pathogenic bacteria are under intense host selection and extreme variation is reported in *lps *specific gene clusters [22]. The observation that the two Xoo strains have different *lps *gene clusters suggests that the plant pathogenic bacteria are also under selection to vary their LPS. Alterations in LPS composition might result in resistance against predators like bacteriophages [4,10] or reduced susceptibility to certain anti-microbial compounds [7] in the host/environment. Most importantly, it might help in evasion of the host defense response.

## Conclusions

These results provide, for the first time, evidence for substantial variation in *lps *biosynthetic gene clusters within different strains of a plant pathogenic bacterium. The results also indicate that multiple HGT events have occurred at this locus in various xanthomonads and provide a new parallel in the mechanisms that plant and animal pathogenic bacteria can employ to generate variability in cell surface molecules.

## Methods

### Complete sequencing of the *lps *locus in the BXO1 strain of Xoo

The *lps *locus was cloned as part of a 35 kb cosmid clone, pSD5. The insert includes 0.6, 2.2, 3.5, 4.0, 6.0, 9.0 and 10 kb fragments upon *Eco*RI (New England Biolabs [NEB], Beverly, MA) digestion and all the fragments were subcloned in to pBlueScript (Stratagene, La Jolla, CA). Most of the sequence obtained in this study was generated by sequencing the 9 kb subclone, pBP4, using a modified shotgun sequencing procedure. Here, pBP4 was digested with *Eco*RI and the 9 kb fragment was gel eluted. Then the fragment was partially digested (1.5–2.5 kb) using a blunt-end cutter, *Hae*III (NEB) and cloned into pMOS (Amersham Pharmacia Biotech, Buckinghamshire, England). The inserts were amplified from random clones by colony PCR using vector primers and were sequenced using an ABI Prism 3700 automated DNA sequencer (Applied Biosystems, Foster City, CA). After editing, the assembly of the sequence data was done using GeneTools (BioTools, Alberta, Canada) and Blast2 [27]. Multiple single strand sequences (3–8 X coverage) were generated for each region in the sequence. Contig assembly was confirmed by restriction fragment analysis of a 12.5 kb PCR amplified product containing the *lps *locus that was obtained using long range PCR (Triple Master™, Eppendorf, Hamburg, Germany) with BXO1 genomic DNA as template. The sizes of the fragments corresponded to the sizes that are predicted by in silico analysis of the sequence (data are not shown). The ORF's were assigned using ORF finder [37] and genes were named as per Bacterial Polysaccharide Genes Nomenclature [20]. Two primers, Pbp1 and Pbp2 (Table 2), were used to derive the sequence of the 0.6 kb *Eco*RI fragment which is also a part of the *lps *locus. The Pbp1 primer binds just after the *wxoC *ORF (which forms part of the 3.5 kb *Eco*RI fragment) and Pbp2 binds within the *wxoD *ORF (which forms part of the 9.0 kb *Eco*RI fragment). A 0.67 kb PCR amplified fragment is obtained from BXO1 genomic DNA using Pbp1 and Pbp2. The band was gel eluted and was sequenced using Pbp1 and Pbp2. The sequence was found to include the 0.6 kb *Eco*RI fragment. In addition, the sequences of all six ORFs were confirmed by sequencing of PCR amplified fragments from genomic DNA using specific sets of gene specific primers (see the list of primers in Table 2).

### Codon Usage Pattern

For each gene the frequency of codon usage for different aminoacids was calculated using a web based program [38]. Further, eight aminoacids i.e., Glycine, Valine, Threonine, Leucine, Arginine, Serine, Proline and Alanine that have atleast four synonymous codons were selected and the percentage of synonymous codons that end with G or C was calculated for each aminoacid and gene. The pattern was calculated for a group of genes by plotting mean values ± SD corresponding to a particular aminoacid. The first group was chosen to include genes that encode proteins which participate in diverse functions and are present at different locations in the Xoo genome outside the *lps *locus. These genes encode: a putative siderophore receptor (AF325732), *Xanthomonas *adhesin like protein (AF288222), a putative phytase (AY151260), *rpfF *(AF411962), shikimate dehydrogenase (AF258797) and secreted xylanase (AF331922). The second group comprised the six genes (excluding transposases) encoded in the Xoo *lps *gene cluster (AF337647).

### Screening of Xoo strains and Xoor for the presence of the genomic island

Specific oligonucleotide primer pairs were designed and used to amplify gene specific fragments for each of the ORFs encoded in the BXO1 genomic island (see the list of primers given in Table 2). DNA sequencing was used to confirm the authenticity of the PCR product obtained with each primer pair using BXO1 genomic DNA as template. Southern hybridizations were performed using these gene specific PCR products as probes. Genomic DNA was isolated from Xoo and Xoor strains according to the procedure described by Leach et. al. [16]. The DNA was then digested with *Eco*RI (NEB) according to supplier's instructions. Digested genomic DNA was separated on a 0.8% agarose gel and vacuum transferred to a Hybond N^+ ^filter (Amersham) using 0.4% NaOH as described by Sambrook et al. [39]. Probes were labelled with α-^32^P dATP using random primer labelling kit (Board of Radiation Technology, Mumbai, India). Prehybridization, hybridisation and washings were done at 68°C as described by Yashitola et al [19]. Membranes were then exposed to phoshoimager plates and images captured using a Fuji FLA-3000 phosphoimager system (Fuji, Japan).

To screen for the presence of the genomic island in different strains, a procedure for colony PCR was standardized. A portion of a single colony (or 10λ of a saturated culture that has approximately 1 × 10^9 ^colony forming units/ml) was lysed in 100λ of 0.01 N NaOH by boiling for 10 minutes. After spinning at 13 K for 1 min., 2λ of supernatant was used as template for PCR using the gene specific primers described above. The products were separated by electrophoresis on 1.5% agarose gels and visualized by ethidium bromide staining.

### TAIL-PCR and sequence analysis

Specific primers were designed against the conserved *metB *and *etfA *gene sequences (Table 2) and the protocol for TAIL-PCR was as originally described by Liu and Whittier [26]. Sequencing of TAIL-PCR products was done using either the cglL3 or etfL3 primer. Homology searches were done using BLAST [21] through NCBI [40] and FASTA [41] through EMBL-EBI [42]. BLAST2 [27] was used to identify the homology break points in the genomic regions that flank the *lps *locus of BXO1 and BXO8. The sequences that were present upstream of the break points were manually examined and three repeat sequences were identified in the 3' coding regions of *metB *and *etfA *genes. Similar repeat sequences were identified in the corresponding regions of BXO8, Nepal624, BXORI, Xac and Xcc. A consensus was derived by aligning these repeat sequences and a particular nucleotide was scored if it is present in a majority of repeats.

### DNA fingerprinting and data analysis

The Xoo IS element, IS*1112 *[16], was used as the hybridisation probe. This probe has been previously used to detect genetic variability in Xoo strains from different countries [15-19]. DNA isolation and Southern hybridisation was done as described in the section on screening of Xoo and Xoor strains for the presence of genomic island. The presence or absence of particular bands was scored as 1 or 0, respectively. The data were analysed using the Dice coefficient option in the program WINDIST [43] to generate distance matrices. The data were used to construct a dendrogram using the NEIGHBOR program in PHYLIP (phylogeny inference software package; University of Washington, Seattle) using the UPGMA (unweighted pair group method of averages) option. To test the robustness of the dendrogram, bootstrap analysis was carried out using the WinBoot program [43] with 2,000 iterations.

### GenBank submissions

The nucleotide sequences obtained in this study have been deposited in GenBank with the following Accession numbers: Sequence of *lps *locus from BXO1 (AF337647); Sequences of TAIL-PCR products from *metB *end of BXO8 (AY319936), Nepal624 (AY319938) and BXORI (AY319940); Sequences of TAIL-PCR products from *etfA *end of BXO8 (AY319937), Nepal624 (AY319939) and BXORI (AY319941).

## Authors' contributions

PBP carried out all the aspects of the work and drafted the manuscript. RVS conceived the study, and participated in its design and coordination. All authors read and approved the final manuscript.

## Figures and Tables

**Table 1:** List of strains used in the study

***Xanthomonas oryzae *pv. *oryzae *(Xoo) strains used in the present study**		
**From India**		
**Strain Name**	**Location**	**Source**
1) BXO1	Chinsuria, West Bengal	Lab collection
2) BXO4	Kapurthala, Punjab	"
3) BXO5	Ferozpur, Punjab	"
4) BXO6	Patiala, Punjab	"
5) BXO7	Titabar, Assam	"
6) BXO8	Nellore, Andhra Pradesh	"
7) BXO13	Marutheru, Andhra Pradesh	"
8) BXO20	Pantnagar, Uttar Pradesh	"
9) BXO479	Nawgam, Gujarat	"
From other countries – (gift from Dr. Jan Leach)		
1) China Xoo NX2		
2) China Xoo #B21		
3) Korea Xoo #197		
4) Korea Xoo #220		
5) Nepal Xoo #537		
6) Nepal Xoo #624		
7) Malaysia Xoo #90		
8) Malaysia Xoo #101		
9) Indonesia Xoo #16		
10)Indonesia Xoo #40		
11)Philippines PXO86		
12)Philippines PXO99A9		
***Xanthomonas oryzae *pv. *oryzicola *(Xoor) strain used in the present study**		
1) BXOR1	Rajendranagar, Andhra Pradesh	lab collection

**Table 2:** Plasmids and primers used in this study

**Plasmids**	**Relevant characteristics**	**Reference or source**
pUFR034	IncW Nm^r ^Mob^+ ^*mob *(P) lacZ alpha Par^+ ^*cos *(8.7 kb)	[44]
Pbluescript	Ap^r^	Stratagene
pSD5	pUFR034 + a 35 kb insert from the BXO1 genome	[8]
pBP1	pBluescript + a 2.2 kb *Eco*RI fragment from pSR1	This study
pBP2	pBluescript + a 3.5 kb *Eco*RI fragment from pSR1	This study
pBP3	pBluescript + a 4.0 kb *Eco*RI fragment from pSR1	This study
pBP4	pBluescript + a 9.0 kb *Eco*RI fragment from pSR1	This study
pBP5	pBluescript + a 10 kb *Eco*RI fragment from pSR1	This study
Primers specific to ORFs present in the genomic island of BXO1		
*WxoA*	Forward primer CCAAGCGACCAGAGGTGCTTGACG	
	Reverse primer GAGGAGCACCATCCGCTACCGCCC	
*WxoB*	Forward primer GTTTTTGTTGGTACTGGGTGCGAG	
	Reverse primer GTACGCCACGGTCAGATCGGCTGC	
*WxoC*	Forward primer CTACTGATGTTGTTCGCAAGGTGG	
	Reverse primer GGCGACTCACCTGCATATCGAGCC	
*WxoD*	Forward primer GTGCTGGTGAGCCATCATTTTG	
	Reverse primer TTACTCACCGGCCATAATCCTTTT	
*Wzt*	Forward primer GACATCGCTATCGAAGTAAAAGGT	
	Reverse primer TCAGGTGCTGTTTGAAGTAGCGGAC	
*Wzm*	Forward primer CATCGGCAAACCCCTTTCGGGT	
	Reverse primer CGCAGCTTACTGATGGAACCCT	
**Primers used for TAIL PCR**		
designed against *metB *gene sequence of BXO1		
CglL1	CTTCGACGCAGCCAAGCGTTTC	
CglL2	CTGCGAGAAGACCGAGCTGTTCAC	
CglL3	CGAATCGCTCGGTGGTGTTGAA	
designed against *etfA *gene sequence of BXO1		
EtfL1	TCGGCCAGACCGGCAAGATCAT	
EtfL2	AGCTGTACATGGCCATCGGCAT	
EtfL3	AGCATCTGACCGGCATCAAGGA	
Other primers described in the paper		
Pbp1	AGCGTGCTGGTGAGCCATCA	
Pbp2	GCAGCAAAAATGCTGTCATAACCA	
